# Spinocerebellar ataxia type 19/22 mutations alter heterocomplex Kv4.3 channel function and gating in a dominant manner

**DOI:** 10.1007/s00018-015-1894-2

**Published:** 2015-04-09

**Authors:** Anna Duarri, Meng-Chin A. Lin, Michiel R. Fokkens, Michel Meijer, Cleo J. L. M. Smeets, Esther A. R. Nibbeling, Erik Boddeke, Richard J. Sinke, Harm H. Kampinga, Diane M. Papazian, Dineke S. Verbeek

**Affiliations:** 1 Department of Genetics, University of Groningen, University Medical Center Groningen, PO Box 30 001, 9700 RB Groningen, The Netherlands; 2 Department of Medical Physiology, University of Groningen, University Medical Center Groningen, 9700 RB Groningen, The Netherlands; 3 Department of Cell Biology, University of Groningen, University Medical Center Groningen, 9700 RB Groningen, The Netherlands; 4 Department of Physiology, University of California at Los Angeles, Los Angeles, CA 90095-1751 USA

**Keywords:** *KCND3*, Kv4.3, Spinocerebellar ataxia, Purkinje cells, Voltage-gated potassium channel

## Abstract

The dominantly inherited cerebellar ataxias are a heterogeneous group of neurodegenerative disorders caused by Purkinje cell loss in the cerebellum. Recently, we identified loss-of-function mutations in the *KCND3* gene as the cause of spinocerebellar ataxia type 19/22 (SCA19/22), revealing a previously unknown role for the voltage-gated potassium channel, Kv4.3, in Purkinje cell survival. However, how mutant Kv4.3 affects wild-type Kv4.3 channel functioning remains unknown. We provide evidence that SCA19/22-mutant Kv4.3 exerts a dominant negative effect on the trafficking and surface expression of wild-type Kv4.3 in the absence of its regulatory subunit, KChIP2. Notably, this dominant negative effect can be rescued by the presence of KChIP2. We also found that all SCA19/22-mutant subunits either suppress wild-type Kv4.3 current amplitude or alter channel gating in a dominant manner. Our findings suggest that altered Kv4.3 channel localization and/or functioning resulting from SCA19/22 mutations may lead to Purkinje cell loss, neurodegeneration and ataxia.

**Electronic supplementary material:**

The online version of this article (doi:10.1007/s00018-015-1894-2) contains supplementary material, which is available to authorized users.

## Introduction

Spinocerebellar ataxia type 19/22 (SCA19/22) is a dominantly inherited neurodegenerative, clinically heterogeneous disorder caused by mutations in *KCND3*, which encodes the voltage-gated potassium channel Kv4.3 [1–3]. Currently, all the SCA19/22 mutations that have been reported lead to a loss of Kv4.3 current amplitude in studies focused on single mutant Kv4.3 subunits [1, 3]. Functional Kv4.3 subunits are assembled as tetrameric complexes and controlled by a large variety of regulatory auxiliary proteins, including KChIPs (potassium channel interacting proteins), which interact with the tetrameric channels to form octomeric complexes, thereby regulating the expression and subcellular localization of the functional subunits (Kv4) and modifying their intrinsic properties [4–6]. Kv4.3 channels exhibit fast activation and inactivation in response to membrane depolarization and recover faster from inactivation than other voltage-gated potassium channels, thus playing an important role in the generation of neuronal transient A-type potassium currents in brain (*I*_A_) [7–12], heart and smooth muscle [7, 13–15]. Neuronal *I*_A_ currents control the spike frequency and the back-propagation of action potentials, processes which are particularly important for Purkinje cell firing [16]. Alterations in Purkinje cell firing could be an early disease manifestation of SCA19/22, as has been observed in other spinocerebellar ataxias (SCA2, SCA3 and SCA27) [17–20]. These alterations may lead to the severe degeneration of Purkinje cells that was found in the atrophic cerebellar vermis of an SCA19 patient [3, 21].

The question remains whether the SCA19/22 mutations result in reduced Kv4.3 channel activity due to haploinsufficiency through pure loss of function and/or due to the mutant proteins acting as dominant negative suppressors of the wild-type Kv4.3 subunits. The latter has been proven for mutations in Kv1.1, which cause episodic ataxia type 1 (EA1) and which display dominant negative effects on potassium currents associated with different clinical phenotypes in EA1 patients [22–26]. Similarly, mutations in Kv3.3 that underlie spinocerebellar ataxia type 13 (SCA13) also seem to result in a loss of function or altered channel gating properties in a dominant manner [27–29]. In this case, depending on the effect of the mutation on Kv3.3 functioning, SCA13 patients exhibit early onset (channel gating deficits) or late onset (loss of channel activity) of the disease. It has also been shown that expression of SCA13 mutant Kv3.3 in Purkinje cells induce cell death by altered excitability or elevated intracellular calcium [30]. Moreover, alterations in the intracellular potassium homeostasis caused by modified Kv signaling can also cause neuronal death [31, 32]. Together, these data imply that Kv channels play an important role in maintaining (cerebellar) neuronal viability.

For SCA19/22, as a dominant disease, we aimed to elucidate whether its pathology is caused by haploinsufficiency through pure loss of Kv4.3 function and/or whether the mutant proteins act as dominant negative suppressors of the wild-type Kv4.3 subunits. We therefore studied wild-type (WT) Kv4.3 trafficking and channel complex formation and function in the presence of SCA19/22-mutant Kv4.3 subunits, to advance our understanding of the underlying disease mechanism(s). All SCA19/22-mutant Kv4.3 subunits exerted dominant negative effects on WT Kv4.3 trafficking leading to intracellular retention and enhanced protein instability of the WT/mutant Kv4.3 channel heterocomplexes that could be rescued by KChIP2. Notably, the trafficking deficit was caused by temperature-sensitive misfolding of the mutant subunits. Only the T352P mutation dominantly reduced the channel activity of WT/T352P heterocomplexes in a dose-dependent manner in *Xenopus* oocytes. In contrast, the ΔF227, M373I and S390N mutations altered the gating kinetics of the WT/ΔF227, WT/M373I and WT/S390N heterocomplexes in a dominant and different manner. Our work demonstrates how difficult it is to establish a common underlying pathological mechanism for SCA19/22, a problem that is reflected in the clinical heterogeneity of this disease. Further work is necessary to reveal the detailed contribution of the mutant Kv4.3 channel subunit in the native *I*_A_ current composition and to elucidate its effects on Purkinje cell functioning.

## Materials and methods

### Molecular biology

The pcDNA3.1-Kv4.3 WT and mutant plasmids was generated as previously described [3], and to generate the ΔF227 mutant, side-directed mutagenesis was performed using the primers forward 5′-GGTGGCCTTCTGCCTGGACACG-3′ and reverse 5′-CGTGTCCAGGCAGAAGGCCACC-3′. To generate Kv4.3 fused to enhanced green fluorescent protein (EGFP), wild-type (WT) and mutant Kv4.3 cDNAs were subcloned into pEGFP-N1 (Clontech) using the *Xho*I and *Eco*RI restriction sites. An extracellular hemagglutinin tag (HA: YPYDVPDYA) was introduced into the cDNA of pcDNA3.1-Kv4.3 wild type (WT) and mutants (T352P, M373I, S390N and ΔF227) in the extracellular loop between transmembrane domains S1 and S2 (L216-P217) using site-directed mutagenesis via forward primer 5′-CCGGGCAGCAAGGAGCTGTACCCATACGACGTCCCAGACTACGCTCCGTGCGGGGAGCGTAC-3′ and reverse primer 5′-GTACGCTCCCCGCACGGAGCGTAGTCTGGGACGTCGTATGGGTACAGCTCCTTGCTGCCCGG-3′, as described previously for Kv4.2. All constructs were verified by Sanger sequencing. The addition of HA and EGFP tags to Kv4.3 did not affect the functional properties of the channel (Fig. S1). The Emerald-KChIP2b was provided by Dr. K. Takimoto (Nagaoka University of Technology, Kamitomoika, Japan) and the cDNA of KChIP2b (referred to as KChIP2) was subcloned in pcDNA3.1 via NcoI.

### Cell culture, transfection and cycloheximide treatment

HeLa cells were grown in Dulbecco’s Modified Eagle’s Medium (Invitrogen) supplemented with 10 % fetal bovine serum (Invitrogen) and 1 % penicillin–streptomycin (Gibco) in a 37 °C incubator with 5 % CO_2_. Transfections were done using polyethylenimine (Polysciences), according to the manufacturer’s instructions. To generate WT/mutant heterocomplex Kv4.3 channels or to generate WT or mutant homocomplex Kv4.3–KChIP2 channels, all plasmids were expressed in 1:1 ratio. Additionally, to generate WT/mutant heterocomplex Kv4.3–KChIP2 channels, plasmids containing WT, mutant Kv4.3 and KChIP2 were transfected in 0.5:0.5:1 ratio. For example, the amount of DNA used for the protein stability experiments was 0.5 µg WT Kv4.3, 0.5 µg mutant Kv4.3 and 1 µg KChIP2 (6 well plate). For the immunocytochemistry and FACS experiments, the amount of plasmid DNA transfected was 0.25 µg WT Kv4.3, 0.25 µg mutant Kv4.3 and 0.5 µg KChIP2 (24 well plate). The protein stability was assessed treating the HeLa cells with a protein synthesis inhibitor cycloheximide (CHX; 25 µg/ml; Sigma) for 0, 3 and 6 h, and protein expression was analyzed by Western blot and quantified as described previously [3].

### Immunological methods

**Fig. 1:**
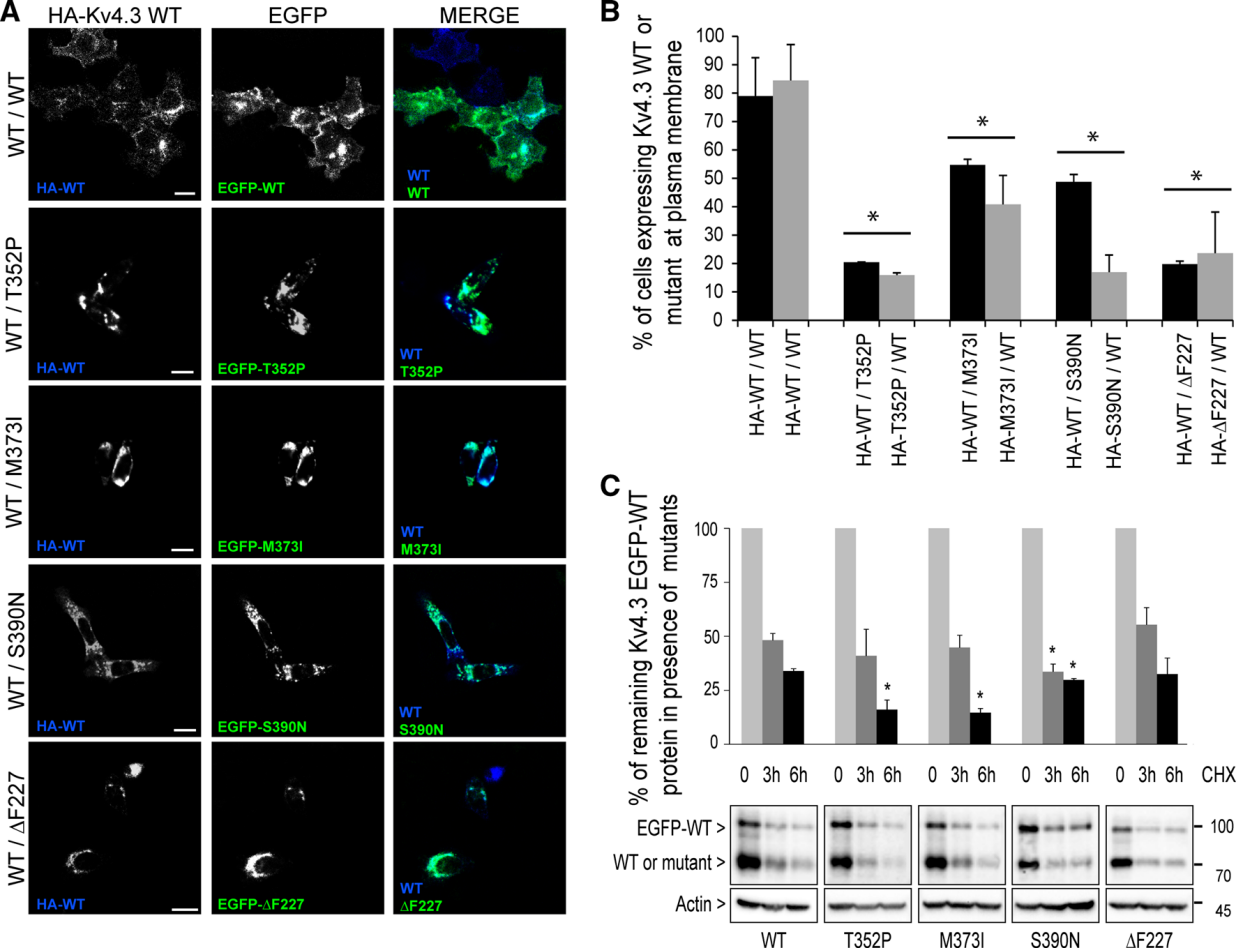
WT/mutant Kv4.3 heterocomplexes are intracellulary retained and less stable than WT/WT Kv4.3 homocomplexes. **a** The confocal images show the anti-HA immuno-staining of permeabilized and fixed HeLa cells co-expressing HA–Kv4.3 WT (*blue*) and EGFP-fused WT, -T352P, -M373I, -S390N or -ΔF227 Kv4.3 (*green*). *Scale bar* 20 µm. **b** Flow cytometry was used to quantify the extracellular HA-tagged Kv4.3 WT (*black bars*) or mutant Kv4.3 (*gray bars*) at the plasma membrane of non-permeabilized HeLa cells expressing either WT/WT homocomplexes or WT/mutant heterocomplexes. The cells expressing Kv4.3 WT on the plasma membrane in the presence of any of the mutant subunits were strongly reduced compared with that in the presence of another WT subunit (*black bars*; WT/WT 79 % vs. WT/T352P: 21 %, WT/M373I: 55 %, WT/S390N: 49 % and WT/ΔF227: 20 %). Likewise, markedly reduced levels of all HA-tagged Kv4.3 mutant subunits were observed in the presence of WT (*gray bars*; WT/WT 84 % vs. T352P/WT: 16 %, M373I/WT: 41 %, S390N/WT: 17 % and ΔF227/WT: 24 %). **c** After 6 h of cycloheximide (CHX) treatment, the WT/mutant heterocomplexes were more rapidly degraded than the WT/WT homocomplexes (remaining protein WT/WT: 34 ± 1.1 %; WT/T352P: 16.1 ± 4.4 %; WT/M373I: 14.7 ± 1.9 %; WT/S390N: 29.7 ± 0.6 %, but not for WT/ΔF227: 32.5 ± 7.4 %). The *bars* represent the normalized expression of WT EGFP-Kv4.3 at *t* = 0 shown in percentages. Data in **b** and **c** represent the average of three independent experiments and the *error bars* represent the mean ± SEM, *t* test and ANOVA *p < 0.05 vs. WT in **b** and *p* < 0.005 vs. WT in **c**

Immunocytochemistry was performed as previously described with some modifications [3]. Briefly, for the complex formation HA–Kv4.3 WT and EGFP-fused Kv4.3 WT or mutants were expressed in HeLa cells (which do not express any Kv regulatory subunit) in the presence or absence of KChIP2. Cells were fixed in 4 % paraformaldehyde in saline phosphate buffer (PBS) for 15 min., permeabilized and blocked in PB buffer (0.1 % Triton X-100, 5 % normal goat serum (NGS) in PBS) for 30 min at room temperature, followed by incubation with the primary antibodies anti-HA (3F10) (1:250; Roche) and anti-KChIP2 (1:250; Abcam) in PBS buffer at 4 °C overnight. Then, cells were washed and incubated with the secondary antibodies goat Alexa 647-conjugated anti-rat and Cy3-conjugated anti-mouse (1:500; Jackson Lab) in PB buffer for 1 h and mounted using Vectashield mounting medium (Vector Labs). For the cell surface detection at 30 °C vs. 37 °C, HeLa cells were transfected with HA-tagged WT or mutant Kv4.3 and cells were cultured at 30 °C or 37 °C for 24 h. Cells were fixed in 4 % paraformaldehyde in PBS for 15 min and blocked with 5 % NGS in PBS for 30 min. Non-permeabilized cells were incubated with anti-HA (3F10) (1:250; Roche) overnight at 4 °C, to detect only the protein at the plasma membrane and goat Alexa 647-conjugated anti-rat. Stacks were acquired using a Leica DMI 6000 Inverted microscope (Leica) equipped with a 40× and 63× oil immersion lens. Images were processed using ImageJ software (National Institutes of Health, Bethesda, MA, USA). The subcellular localization of the WT/mutant heterocomplex Kv4.3 channels with and without KChIP2 is also depicted as cell fluorescence intensity plot profiles (Fig. S2). These figures combine the outcomes of the immunofluorescence experiments as shown in Figs. 1 and 3. In addition, the addition of cycloheximide to cells transfected with the various heterocomplex channels did not change the cellular localization of the channel complexes (Fig. S3).

For the cell surface quantification using flow cytometry of the WT/mutant heterocomplex Kv4.3 channels, HeLa cells were transfected with a combination of extracellular HA-tagged WT with EGFP-tagged Kv4.3 WT or mutants, and HA-tagged mutant with EGFP-tagged Kv4.3 WT, either with or without KChIP2. For quantification of cell surface expression of WT or mutant Kv4.3 subunits at 30 vs. 37 °C, HeLa cells were transfected with HA–WT or HA–mutant Kv4.3 together with GFP (used to quantify the total amount of transfected cells) and cultured at 30 or 37 °C for 24 h. Cells expressing WT Kv4.3 together with WT-Kv4.3-HA were used as positive control and non-transfected cells were used as a negative control. Non-permeabilized cells were incubated with anti-HA (3F10) antibody (1:500, Roche) for 2 h at 37 °C or at 30 °C and secondary Alexa647-conjugated anti-rat antibody (1:500, Jackson Lab). After washing, cells were re-suspended in 500 µl ice-cold 10 % fetal bovine serum in PBS. Cytometric analysis was performed using an FACS Calibur flow cytometer (BD Biosciences) with 488 and 635 nm lasers, and analyzed using the Cell Quest Pro software (BD Biosciences). Representative images for the FACS analysis are shown in Fig. S4.

For protein analysis, cells were homogenized in 2 % SDS-PBS buffer containing protease inhibitor cocktail (Roche) and protein concentration was determined using the BCA method (BioRad). Then, 50 µg of protein were separated in an SDS-PAGE, blotted in nitrocellulose membrane, blocked and incubated with mouse anti-Kv4.3 (K75/41; NeuroMab; 1:1000), mouse anti-actin (MP Biochemicals; 1:5000) and mouse anti-KChIP2 (Abcam; 1:1000). Final quantification was performed using the program Quantity One (Bio-Rad).

### Electrophysiology in oocytes

The *Xenopus laevis* oocyte expression system was used to characterize the functional properties of WT and mutant Kv4.3 channels in the presence of KChIP2. All animal procedures were approved by the Chancellor’s Animal Research Committee at the University of California, Los Angeles. RNA was transcribed in vitro using the mMessage mMachine T7 Ultra kit (Ambion) and injected into stage V–VI oocytes. RNAs (160–200 ng) encoding WT or mutant Kv4.3 or a mixture of WT and mutant Kv4.3 were co-injected with RNA encoding KChIP2, which was included at equimolar amounts to WT Kv4.3. The oocytes were kept in an 18 °C incubator and all oocyte recordings were performed at room temperature (~18 °C). Potassium currents were recorded 1–3 days later using a Warner OC-725 two-electrode voltage clamp, as described previously with some modifications [27, 33, 34]. Electrodes were filled with 3 M KCl and had resistances ranging from 0.3 to 1.0 MΩ. The bath solution contained 2 mM KCl, 96 mM NaCl, 0.5 mM CaCl_2_ and 5 mM HEPES, pH 7.5. Alternatively, in some experiments the bath solution contained 2 mM KCl, 96 mM NaCl, 1.8 mM CaCl_2_, 1 mM MgCl_2_ and 5 mM HEPES, pH 7.5 (Fig. S5). Linear leak and capacitive currents were subtracted using a P/-4 protocol. The conductance/voltage relationship was determined by pulsing the membrane from a holding potential of −100 mV to voltages ranging from −80 to +70 mV in 10 mV increments. Conductance values were calculated from peak current amplitudes assuming a linear open channel current–voltage relationship and a reversal potential of −95 mV, and normalized to the maximum value obtained in the experiment. Normalized conductance values were plotted versus voltage and fitted with a single Boltzmann function to obtain values for *V*_½,act_ and the slope factor. Mean current density plots were generated by measuring the peak current amplitudes as a function of voltage and normalized to that for wild-type Kv4.3 expressed in parallel in the same batch of oocytes. Data from different batches of oocytes were then averaged. RNA encoding KChIP2 was co-injected at an equimolar ratio with the total amount of Kv4.3 RNA.

To characterize the steady-state properties of inactivation, the voltage was stepped from the holding potential (−100 mV) to prepulse potentials ranging from −130 to +10 mV for 1 s to allow inactivation to occur. The membrane was then stepped to the test potential of +60 mV for 1 s. The extent of inactivation during the prepulse was calculated as the ratio of peak current amplitude during the test pulse relative to the peak current amplitude at +60 mV in the absence of a prepulse (*I*/*I*_max_). Data were plotted as a function of prepulse voltage and fitted with a Boltzmann function to determine the midpoint voltage, *V*_½,inact_ and the slope factor. The kinetics of recovery from inactivation at −80 mV were assessed using a two-pulse protocol, in which two pulses to +60 mV were applied separated by an interpulse interval of varying duration at −80 mV. The fractional recovery was calculated as the ratio of the peak current amplitude during the second pulse to that of the first pulse (*I*/*I*_max_). This ratio was plotted versus the interpulse duration. The time to reach peak amplitude was measured by evoking currents at +60 mV, which were then scaled to the same amplitude and overlaid. Additionally, the fraction of current remaining at the end of the pulse was measured. Data were fitted with one exponential component to estimate the recovery time constant, *τ*_rec_.

### Statistical analysis

The data obtained from the flow cytometry and Western blot densitometries were analyzed by one-way analysis of variance (ANOVA) followed by Student’s *t* test. All data shown are representative for at least three independent experiments and presented as mean ± SEM. The electrophysiology experiments were analyzed with ANOVA followed by Student’s *t* test. *P* values less than 0.05 were considered significant.

## Results

### Kv4.3 mutants display a dominant negative effect on the trafficking and stability of the wild-type protein

We and others have previously shown that the T352P, M373I, S390N and ΔF227 mutant homocomplexes exhibit impaired trafficking and/or stability [1, 3]. Since SCA19/22 patients carry one WT and one mutant *KCND3* allele, we wondered whether the mutant (T352P, M373I, S390N and ΔF227) and WT Kv4.3 subunits would assemble into functional tetrameric WT/mutant heterocomplexes at the plasma membrane. We first studied the effect of the mutant subunits on the protein trafficking of WT Kv4.3. To study the subcellular localization of the WT/mutant heterocomplexes, HA-tagged WT Kv4.3 was co-expressed with either enhanced green fluorescent protein (EFGP)-fused T352P, M373I, S390N, ΔF227 or WT Kv4.3 in HeLa cells. Immunocytochemistry revealed that, in the presence of any mutant subunit, WT Kv4.3 was mainly detected intracellularly co-localizing with the mutant subunit, and almost no plasma membrane localization was seen (Fig. 1a). In contrast, WT/WT homocomplexes showed abundant plasma membrane localization. Flow cytometric analysis was used to quantify the presence of the extracellular HA-tagged WT or mutant Kv4.3 at the plasma membrane of non-permeabilized cells. These results confirmed the strongly reduced WT Kv4.3 signal at the plasma membrane in the presence of any mutant subunit (Fig. 1b, black bars), coinciding with similarly low levels of mutant Kv4.3 at the cell surface in the presence of WT (Fig. 1b, gray bars).

Since the ER-retained mutant proteins were previously shown to be unstable [3], we next assessed the protein stability of WT Kv4.3 in the presence of the mutant subunits. A time course cycloheximide (CHX) experiment was performed in HeLa cells expressing WT EGFP-Kv4.3 together with either WT or mutant subunits. The fraction of protein remaining was analyzed by Western blot and quantified, revealing that despite similar initial protein levels, after 6 h of CHX treatment, WT Kv4.3 degraded significantly faster in the presence of T352P, M373I and S390N mutants than in the presence of ΔF227 or another WT subunit (Fig. 1c). The change in the cellular localization of the WT/mutant heterocomplex Kv4.3 channels was not due to the reduced protein stability of these complexes (Fig. S3). These data clearly indicate that all mutant subunits have a dominantly negative effect on the trafficking of WT Kv4.3 to the plasma membrane, resulting in intracellular retention of WT/mutant heterocomplexes. Furthermore, the data show that T352P, M373I and S390N (but not ΔF227) cause reduced protein stability of the heterocomplexes in a dominant manner.

### SCA19/22-mutant Kv4.3 subunits exhibit a temperature-sensitive folding deficit

**Fig. 2:**
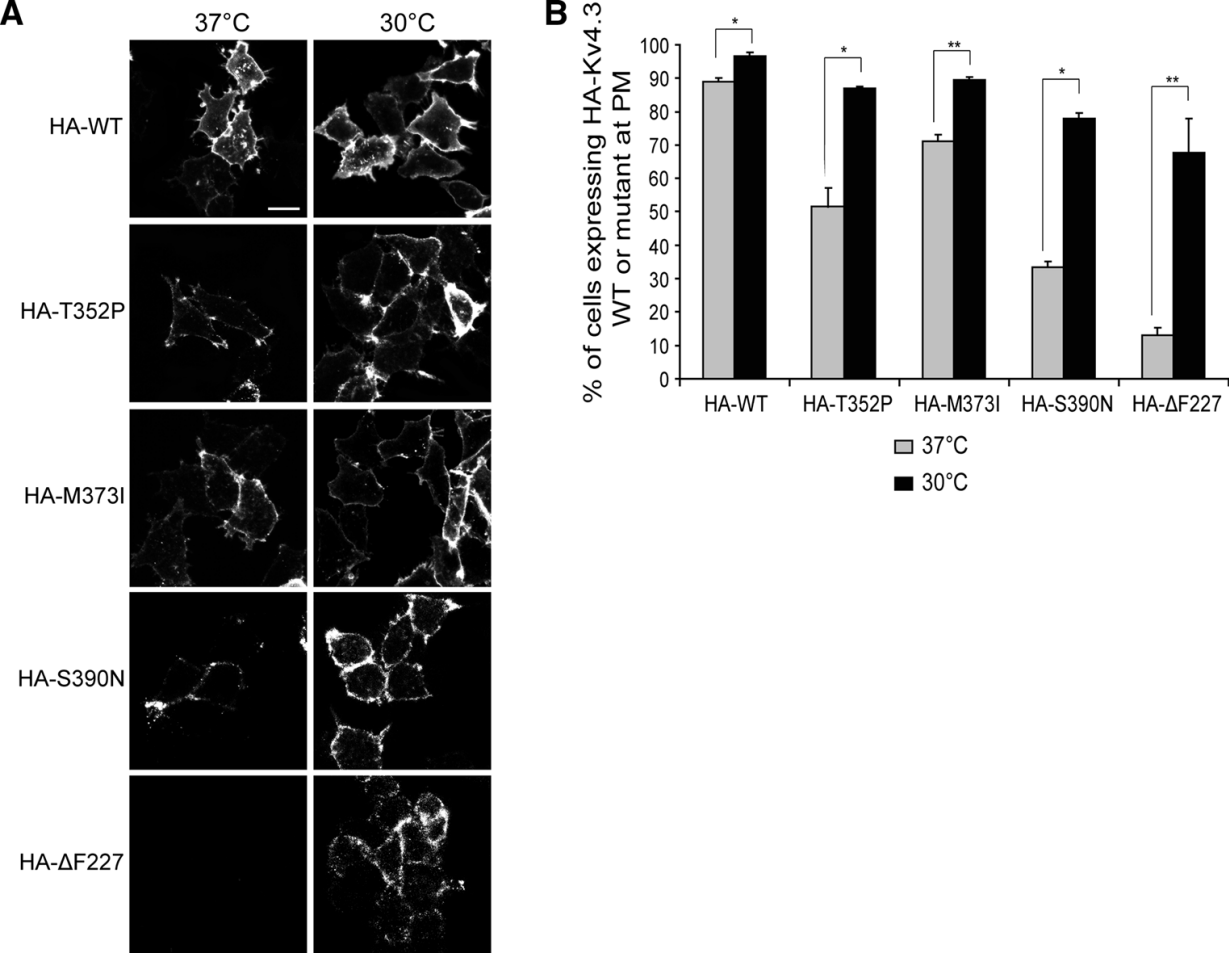
Kv4.3 mutant subunits exhibit a temperature-sensitive folding defect. **a** Images of HeLa cells expressing HA–Kv4.3 WT or HA–T352P, –M373I, –S390N and –ΔF227 incubated at 37 °C or at 30 °C for 24 h. Non-permeabilized cells were stained using anti-HA antibody to detect Kv4.3 at the plasma membrane. *Scale bar* 20 µm. **b** Flow cytometry analysis is used to quantify the level of HA–Kv4.3 at the cell surface in HeLa cells cultured at 37 °C or at 30 °C. The *graphs* show a significant increase in the number of cells expressing the mutant subunits at the plasma membrane cultured at 30 °C (*black bars*; WT: 89 % ± 1.8; T352P: 86.9 % ± 1.2; M373I: 89.5 % ± 1.4; S390N: 77.8 % ± 3.3; and ΔF227: 67.8 % ± 10.1) compared to cells cultured at 37 °C (*gray bars*; WT: 96.5 % ± 2.1; T352P: 55.4 % ± 5.5; M373I: 71.1 % ± 3.4; S390N: 33.4 % ± 3; and ΔF227: 13.1 % ± 0.6). *Bars* show the average of three independent experiments (mean ± SEM, *p < 0.01, **p < 0.001)

Additionally, we tested if the trafficking defect observed for the SCA19/22 mutants was temperature sensitive as this has been seen before for other mutant channels, including ΔF508 mutant cAMP-regulated chloride channels (CFTR) [35–37] or N470D mutant Kv11.1 channels [38, 39]. To improve the protein trafficking of mutant Kv4.3 to the plasma membrane, HeLa cells expressing either WT Kv4.3 or T352P, M373I, S390N and ΔF227 mutant Kv4.3 containing an extracellular HA-tag were incubated at 30 °C followed by immunofluorescence analysis. Notably, culturing at 30 °C led to markedly increased levels of all the mutant Kv4.3 homocomplex channels at the plasma membrane (Fig. 2a, right panel), whereas cells cultured at 37 °C had significantly lower levels at the plasma membrane compared with WT Kv4.3 (Fig. 2a, left panels). We pursued this temperature effect further using flow cytometry to quantify the percentage of cells expressing Kv4.3 channel complexes at the plasma membrane and confirmed that cell surface expression of all the mutant channel complexes was significantly increased at 30 °C compared to 37 °C (Fig. 2b). Given that low-temperature culturing restored proper plasma membrane localization of all mutant channel complexes, our findings indicate that SCA19/22 mutations in Kv4.3 induce a temperature-sensitive folding/trafficking defect.

### KChIP2 drives stable and correctly assembled WT/mutant heterocomplex Kv4.3 channels at the plasma membrane

**Fig. 3:**
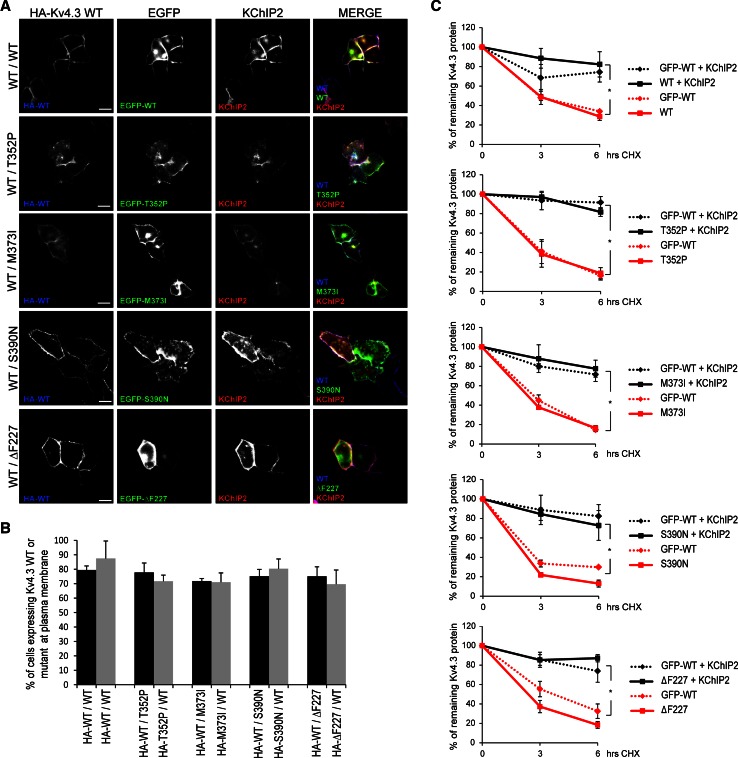
KChIP2 drives the formation of stable WT/mutant Kv4.3 heterocomplexes at plasma membrane. **a** Confocal images of permeabilized and fixed HeLa cells expressing KChIP2 together with HA–WT/EGFP–WT homocomplexes or HA–WT/EGFP–mutant heterocomplexes (*green*) stained with anti-HA (*blue*) and anti-KChIP2 (*red*) antibodies. In the presence of KChIP2, all WT/mutant heterocomplexes were detected at the plasma membrane (merged in *violet*). *Scale bar* 20 µm. **b** Flow cytometry was used to quantify the percentage of cells expressing the extracellular HA-tagged Kv4.3 WT (*black bars*) or mutant subunits (*gray bars*) in the presence of KChIP2 at the plasma membrane of non-permeabilized HeLa cells. Similar levels of HA–Kv4.3 WT were detected at the plasma membrane for WT/WT homocomplexes vs. WT/mutant heterocomplexes (WT/WT: 79.5 % vs. WT/T352P: 77.8 %, WT/M373I: 71.8 %, WT/S390N: 75.1 % and WT/ΔF227: 75 %). Similarly, the levels of HA-tagged Kv4.3 mutants at plasma membrane for mutant/WT heterocomplexes were similar to WT/WT homocomplexes (WT/WT: 87.5 % vs. WT/T352P: 71.6 %, WT/M373I: 71 %, WT/S390N: 80.3 % and WT/ΔF227: 69.6 %). **c** Time course cycloheximide (CHX) experiments were performed in HeLa cells expressing the EGFP-WT/WT homocomplexes or EFGP–WT/mutant heterocomplexes in the presence (*black lines*) or absence (*red lines*) of KChIP2, and the remaining protein was analyzed by Western blot and quantified. Notably, in the presence of KChIP2, all WT/mutant heterocomplexes were significantly more stable (*black lines*) than the heterocomplexes without KChIP2 (*red lines*). Data in **b** and **c** represent the average of three independent experiments and the *error bars* represent the mean ± SEM, *t* test in **b** showed no significant differences and *p < 0.00001 vs. KChIP2 presence in **c**. In **c** the graphs represent the Western blot protein densitometries normalized by actin, showing the percentage of the remaining Kv4.3 protein

We have previously shown that KChIP2 was able to rescue the trafficking defects and protein instability of the single T352P and M373I subunits. However, KChIP2 was unable to rescue these deficits for S390N [3] and ΔF227 channels (unpublished data). We therefore wondered whether KChIP2 could also rescue the intracellularly trapped WT/mutant heterocomplexes. To examine whether this is the case, we expressed KChIP2 with HA–WT Kv4.3 and EGFP-mutant subunits in a 1:1 ratio in HeLa cells. KChIP2 was able to rescue the trafficking deficits of all WT/mutant heterocomplexes, including the complexes containing S390N or ΔF227 subunits, as shown by immunocytochemistry (Fig. 3a; Fig. S2). Flow cytometry analysis confirmed the presence of both HA–WT and HA–mutant Kv4.3 subunits in similar levels at the cell surface in the presence of KChIP2 (Fig. 3b), indicative of a properly assembled Kv4.3 WT/mutant heterocomplex. Additionally, the protein stability of the WT/mutant heterocomplexes was assessed using a time course CHX experiment in HeLa cells in the presence or absence of KChIP2. Our data show that KChIP2 also prevented the rapid degradation of the WT/mutant heterocomplexes (Fig. 3c), restoring the protein stability to the same levels of WT/WT homocomplexes.

These data show that KChIP2 can eliminate the dominant negative effect of the mutant subunits on the trafficking and stability of the WT/mutant heterocomplexes. Thus, in the presence of sustainable levels of KChIP2, stable and correctly assembled WT/mutant heterocomplex Kv4.3 channels are located at the plasma membrane in a heterologous system.

### T352P mutant Kv4.3 suppresses WT Kv4.3 activity in *Xenopus* oocytes, while M373I, S390N and ΔF227 mutant subunits alter channel properties in a dominant manner

Since correctly assembled macromolecular heterocomplexes consisting of KChIP2, WT and mutant Kv4.3 subunits are present at the plasma membrane, we wondered whether these channel complexes were functionally active. The *Xenopus* oocyte expression system was used to analyze the strength of any dominant effects of the mutant Kv4.3 subunits and to determine the effects of the mutations on the functional properties of WT/mutant heterocomplexes. Advantageously, oocytes are kept at 18 °C, which could enhance the rescue of temperature-dependent folding and trafficking defects in a manner similar to that observed for HeLa cells at 30 °C.

**Fig. 4:**
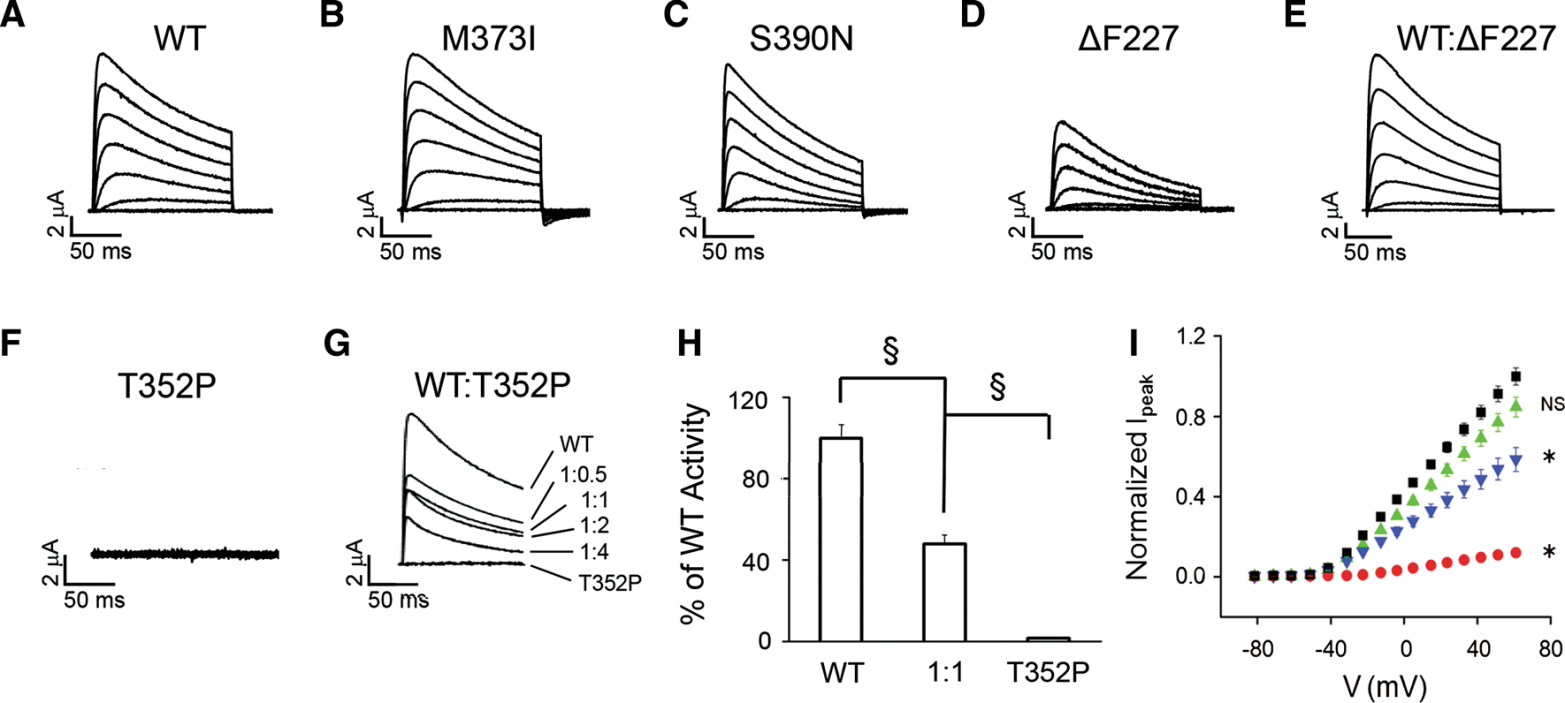
T352P-mutant Kv4.3 suppresses Kv4.3 WT activity by a dominant mechanism. **a**–**f** Representative current traces, recorded in oocytes at ~18 °C with a two-electrode voltage clamp 1–3 days after RNA injection, are shown for **a** WT, **b** M373I, **c** S390N, **d** ΔF227, **e** a 1:1 mixture of WT and ΔF227 and **f** T352P. Currents were evoked by pulsing from a holding potential of −100 mV to voltages ranging from −80 to +70 mV in 10 mV increments. For clarity, every other trace has been omitted. **g** Representative current traces evoked at +60 mV are shown for oocytes injected with WT, T352P or indicated ratios of WT:T352P RNA have been overlaid. **h** WT or T352P were expressed separately or at a 1:1 ratio keeping the amount of WT RNA constant. Normalized peak current amplitudes measured at +60 mV were WT alone, 1.00 ± 0.06 (*n* = 24); WT:T352P at a 1:1 ratio, 0.48 ± 0.09 (*n* = 30); and T352P alone, 0.02 ± 0.01 (*n* = 7). The 1:1 ratio differed significantly from WT alone, evaluated using a one-way ANOVA followed by Student’s *t* test (^§^ *p* < 0.00001). **i** Current density plot. Normalized peak current amplitude as a function of voltage is shown for wild type (*black squares*), ΔF227 (*red circles*), wild type:ΔF227 expressed at a 1:1 molar ratio (*green triangles*) or wild type:T352P expressed at a 1:1 molar ratio (*blue inverted triangles*). RNA encoding KChIP2 was co-injected at an equimolar ratio with the total amount of Kv4.3 RNA. Peak current amplitudes were measured as a function of voltage and normalized to that for wild-type Kv4.3 expressed in parallel in the same batch of oocytes. Data from different batches of oocytes were then averaged (*n* = 12). Data are provided as mean ± SEM. Statistical significance was assessed using data obtained at +50 mV: *significantly different from wild type (*p < 0.05) by ANOVA followed by Tukey’s post hoc test; *NS* not significantly different from wild type

For functional analysis in oocytes, RNA encoding WT Kv4.3, mutants or an equal mixture of WT and mutant was co-injected with KChIP2 RNA (1:1). Electrophysiological experiments were performed at ~18 °C. Representative current traces, 150 ms in duration, are shown in Fig. 4a–f. Compared with WT Kv4.3 (Fig. 4a), M373I and S390N homocomplexes resulted in robust K^+^ currents (Fig. 4b, c) and the ∆F227 subunit produced active channels, but with significantly reduced current amplitudes (Fig. 4d). Current amplitudes were increased when the ∆F227 subunit was coexpressed at a 1:1 ratio with WT subunits (Fig. 4e). In contrast, no channel activity was detected for T352P homocomplexes (Fig. 4f). To determine if there was any dominant negative effect of the mutant subunit, T352P was co-expressed with WT at various ratios. This led to a marked dose-dependent suppression of current amplitude compared with the current amplitude of WT homocomplexes (Fig. 4g, h). In contrast, WT/M373I and WT/S390N heterocomplexes produced active channels (Table 1). To further characterize the effects of the mutations on current amplitude, mean current density plots were generated for WT, ΔF227, WT/ΔF227 and WT/T352P channel complexes (Fig. 4i). Normalized peak current amplitudes were significantly reduced for WT/T352P heterocomplexes and ∆F227 homocomplexes. Thus, T352P exerts marked dominant suppression of WT channel activity. ΔF227 forms active channels less efficiently than WT, but peak current amplitude was restored to the WT level when ∆F227 was co-expressed with WT subunits. M373I and S390N form fully active homocomplexes and WT/mutant heterocomplexes.

**Table 1:** Functional properties of WT and SCA19/22-mutant Kv4.3 channels

	Activation		Time to peak (ms)	*I*_150ms_/*I*_peak_	Inactivation		
	*V*_½,act_ (mV)	Slope (mV)			*V*_½,inact_ (mV)	Slope (mV)	*τ*_rec_ (ms)
Kv4.3 WT	−20 ± 1 (*29*)^a^	17 ± 0.3 (*29*)	9.9 ± 0.4 (*29*)	0.47 ± 0.02 (*29*)	−58 ± 1 (*24*)	5 ± 0.1 (*24*)	244 ± 15 (*18*)
ΔF227	7 ± 1 (*18*)^§,b^	18 ± 1 (*18*)	12.6 ± 0.7 (*18*)^#^	0.25 ± 0.02 (*18*)^§^	−50 ± 1 (*12*)^§^	6 ± 0.3 (*12*)^§^	108 ± 5 (*9*)^§^
WT:ΔF227 (1:1)	−11 ± 1 (*14*)^§^	18 ± 1 (*14*)	10.0 ± 0.3 (*13*)	0.42 ± 0.01 (*13*)	−57 ± 1 (*15*)	5 ± 0.1 (*15*)	206 ± 20 (*15*)
T352P^c^	ND	ND	ND	ND	ND	ND	ND
WT:T352P (1:1)	−20 ± 1 (*24*)	18 ± 1 (*24*)	7.4 ± 0.4 (*23*)^§^	0.39 ± 0.02 (*23*)^‡^	−60 ± 1 (*14*)	5 ± 0.3 (*14*)	245 ± 14 (*7*)
M373I	−20 ± 1 (*12*)	15 ± 1 (*12*)^‡^	12.0 ± 0.3 (*12*)^‡^	0.49 ± 0.01 (*12*)	−53 ± 2 (*13*)^‡^	5 ± 0.2 (*13*)	279 ± 17 (*4*)
WT:M373I (1:1)	−18 ± 1 (*5*)	14 ± 2 (*5*)*	12.8 ± 0.1 (*5*)^‡^	0.39 ± 0.03 (*5*)	−54 ± 1 (*6*)^‡^	5 ± 0.3 (*6*)	ND
S390N	−19 ± 1 (*10*)	19 ± 1 (*10*)*	6.8 ± 0.5 (*10*)^#^	0.34 ± 0.01 (*10*)^#^	−63 ± 2 (*10*)^‡^	4 ± 0.1 (*10*)*	194 ± 14 (*4*)
WT:S390N (1:1)	−17 ± 2 (*2*)	19 ± 1 (*2*)	7.5 ± 0.9 (*2*)	0.24 ± 0.03 (*2*)	−63 ± 1 (*4*)^‡^	6 ± 1 (*4*)	ND

In all cases, KChIP2 RNA was co-expressed in 1:1 molar ratio with WT Kv4.3 RNA

*ND* not done

^a^Values are provided as mean ± SEM (*n* = number of measurements)

^b^Statistical significance compared with WT was evaluated by one-way ANOVA followed by Student’s *t* test: * *p* < 0.05; ^‡^ *p* < 0.005; ^#^ *p* < 0.0005; ^§^ *p* < 0.00001

^c^T352P did not produce functional channels when expressed alone

**Fig. 5:**
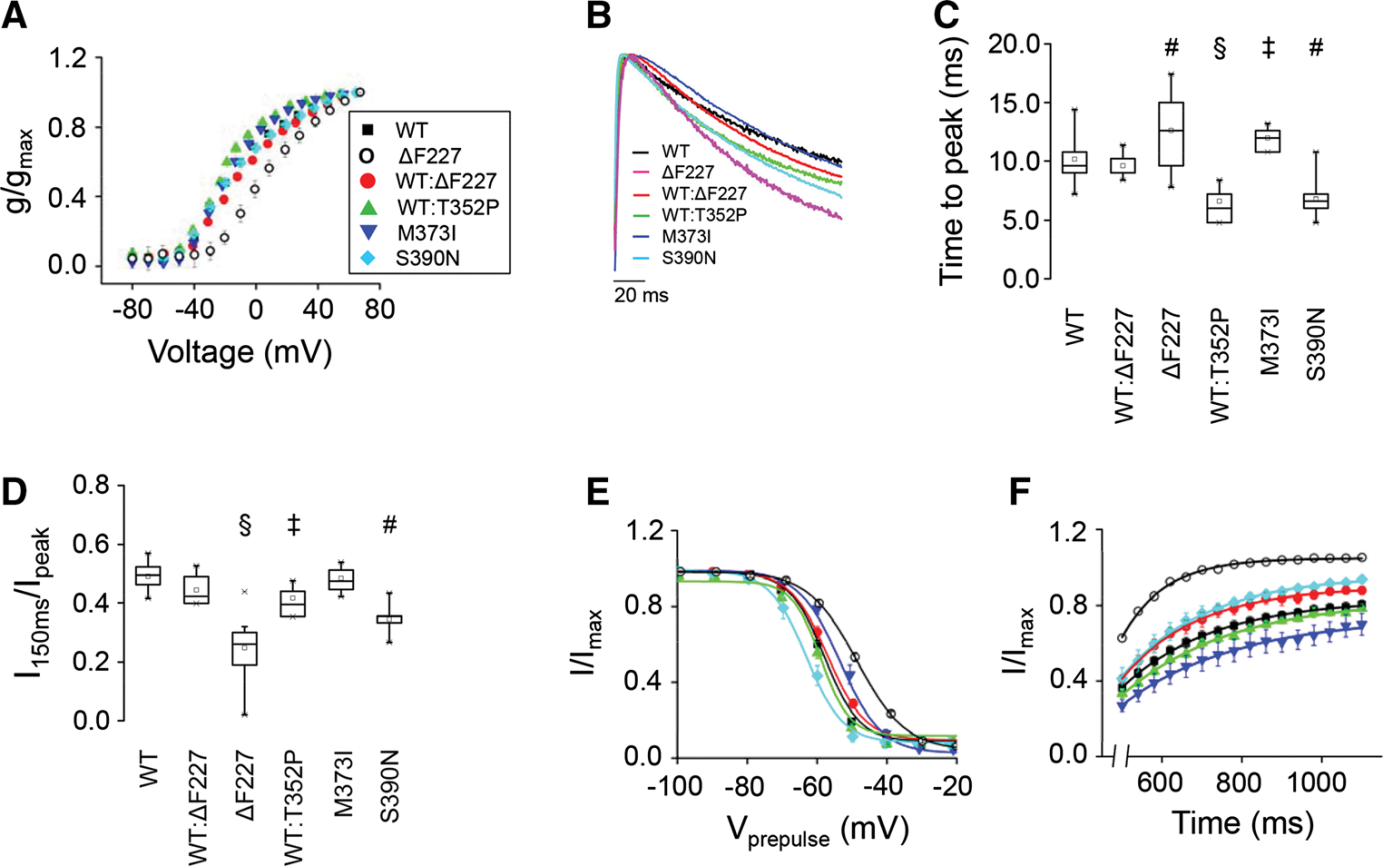
SCA19/22 mutations alter the functional properties of Kv4.3 homocomplexes and heterocomplexes. **a** Conductance values were calculated from peak current amplitudes assuming a linear open channel current–voltage relationship and normalized to the maximum value obtained in the experiment. Normalized conductance values were plotted as a function of test voltage for WT (*black squares*), ΔF227 (*open circles*), a 1:1 mixture of WT and ΔF227 (*red circles*), a 1:1 mixture of WT and T352P (*green triangles*), M373I (*blue inverted triangles*) and S390N (*diamond symbols*) and data were fitted with a single Boltzmann function to obtain values for *V*_½,act_ and the slope factor (see Table 1). **b** Representative current traces evoked by pulsing from −100 to +60 mV have been scaled and overlaid for WT (*black*), ΔF227 (*magenta*), a 1:1 mixture of WT and ΔF227 (*red*), a 1:1 mixture of WT and T352P (*green*), M373I, (*blue*) and S390N (*cyan*). **c** The *box plot* shows the time to reach peak current amplitude at +60 mV. **d** The *box plot* shows the *I*_150ms_/*I*_peak_ ratio, calculated by dividing the current amplitude remaining at the end of a 150 ms pulse by the peak current amplitude. **e** Steady-state inactivation was evaluated using a two-pulse protocol. Peak amplitudes during the test pulse were normalized to the peak amplitude in the absence of a prepulse (*I*/*I*_max_) and plotted versus prepulse voltage. Data were fitted with a single Boltzmann function (*solid curves*) to obtain values for *V*_½,inact_ and the slope factor (see Table 1). **f** To measure the rate of recovery from inactivation, currents were evoked by pulsing from −80 to +60 mV for 400 ms (pulse 1). The voltage was then returned to −80 mV for variable durations ranging from 500 to 1100 ms, in 40 ms steps, prior to a second pulse to +60 mV for 400 ms (pulse 2). The fractional recovery was calculated as the peak amplitude during pulse 2 divided by the peak amplitude during pulse 1 (*I*/*I*_max_) and plotted versus the interpulse duration. Each data set was fitted with a single exponential function to obtain the time constant for recovery (*τ*_rec_) (Table 1). Data are shown as mean ± SEM. In **b** and **c**, statistical significance compared to WT alone was evaluated by one-way ANOVA followed by Student’s *t* test: ^‡^ *p* < 0.005; ^#^ *p* < 0.0005; ^§^ *p* < 0.00001. Mean values ± SEM of the time to peak and the *I*_150ms_/*I*_peak_ ratio are provided in Table 1. Results obtained with 1:1 mixtures of WT:M373I and WT:S390N are provided in Table 1

In all cases, K^+^ currents showed rapid activation and subsequent inactivation characteristic of Kv4.3 channels. In our experiments, inactivation was slower than in previous reports, because currents were recorded at ~18 °C rather than the more typical 22 °C (Fig. S4). At the lower temperature, a slow component of inactivation dominates the kinetics of current decay, whereas at 22 °C fast and slow components make similar contributions to decay kinetics (Table S1). We compared the steady-state properties of activation in WT and mutant homo- and heterotetrameric channels by determining peak conductance/voltage relationships (Fig. 5a). The data sets were fitted with single Boltzmann equations to obtain values for the voltage at which 50 % of the channels are activated (*V*_½,act_) and the slope factor (Table 1). The ∆F227 homocomplexes significantly shifted the value of V_½,act_ by +27 mV in the depolarized direction and the activation of WT/∆F227 (1:1) heterocomplexes was shifted by +9 mV (Fig. 5a; Table 1), a value intermediate to WT and ∆F227 alone. In contrast, the other mutant homo- and heterocomplexes had *V*_½,act_ values similar to those of WT. Additionally, M373I homocomplexes and WT/M373I (1:1) heterocomplexes had significantly reduced slope factors compared to WT (Table 1), and S390N had small but statistically significant effects on the slope factor of the activation curve, increasing the value by 2 mV, indicative of a partial dominant effect on the steady-state activation.

To quantitatively compare the kinetic properties of activation and inactivation in mutant and WT channels, we measured the time to reach peak current amplitude and the ratio of current amplitude measured at the end of a 150 ms pulse to +60 mV relative to the peak current amplitude (*I*_150ms_/*I*_peak_) (Fig. 5b–d; Table 1). The time to reach peak current amplitude reflects the competing processes of activation and inactivation. Therefore, changes in the time to peak may reflect changes in the rate of activation, inactivation or some combination of the two. Both ΔF227 and M373I homocomplexes significantly increased the time to peak compared to WT homocomplexes, whereas S390N channels reached the peak amplitude faster (Fig. 5b, c; Table 1). In contrast, the time to peak in WT/ΔF227 and WT/S390N (1:1) heterocomplexes did not differ significantly from WT expressed alone, while in WT/T352P channels the time to peak was significantly decreased (Fig. 5b, c; Table 1). The *I*_150ms_/*I*_peak_ ratio was significantly decreased in ΔF227, S390N homocomplexes and WT/T352P (1:1) heterocomplexes compared to WT alone, consistent with the idea that these mutations increase the rate of inactivation (Fig. 5d; Table 1). In contrast, the *I*_150ms_/*I*_peak_ ratio was unchanged in M373I homocomplexes and WT/ΔF227 (1:1) heterocomplexes (Fig. 5d; Table 1).

The effects of the mutations on the voltage dependence and extent of inactivation were analyzed using a two-pulse protocol. The data sets were plotted as a function of prepulse voltage and fitted with Boltzmann functions to determine the midpoint voltage, *V*_½,inact_, at which 50 % of the channels are inactivated and the slope factor (Fig. 5e; Table 1). For the ΔF227 and M373I homocomplexes, *V*_½,inact_ shifted toward the depolarized direction. In contrast, for S390N homocomplexes, *V*_½,inact_ shifted in the hyperpolarized direction. Notably, WT/ΔF227 (1:1) heterocomplexes did not show changes in the voltage dependence of inactivation and behaved like WT homocomplexes, whereas WT/M373I and WT/S390N (1:1) heterocomplexes exhibited the same shift in inactivating voltage as the respective mutant homocomplex channels. Alterations in the slope factor were only detected for ΔF227 and S390N homocomplexes (Table 1). These data indicate that only the M373I and S390N mutations exhibit dominant effects on steady-state inactivation, whereas T352P and ΔF227 do not.

Lastly, we assessed the kinetics of recovery from inactivation, by calculating the ratio of the peak amplitude of two separate pulses (*I*/*I*_max_), and the *I*/*I*_max_ was plotted versus the interpulse duration (Fig. 5f). Note that the curves in Fig. 5f are offset from each other due to differences in the extent of inactivation during the first pulse. Data were fitted with one exponential component to estimate the recovery time constant, *τ*_rec_ (Table 1). Notably, only ΔF227 homocomplexes significantly decreased *τ*_rec_, indicating that the ΔF227 mutation increased the rate of recovery from inactivation (Fig. 5f; Table 1). But, again, WT/ΔF227 heterocomplexes (ratio 1:1) behaved like WT homocomplexes, indicating the absence of a dominant effect for this end point as well. In contrast, none of the other mutations affected the rate of recovery from inactivation.

## Discussion

We have clearly shown that all SCA19/22-mutant Kv4.3 subunits exhibit dominant effects on WT protein function and revealed that both haploinsufficiency by loss of function and dominant (negative) effects may contribute to the disease etiology. We consider three potential scenarios leading to partial haploinsufficiency and/or dominant (negative) effects due to SCA19/22 mutations. In interpreting our data, we assume that the formation of WT/mutant heterocomplexes is a stochastic process in which WT and mutant subunits assemble randomly in tetrameric complexes when they are expressed in a 1:1 ratio. Additionally, we assume that the stoichiometry of Kv4.3/KChIP2 complex is variable and depends on the expression levels of KChIP2. This was supported by a recent publication that showed that the stoichiometry of Kv4.2/KChIP4 complex depends on the expression level of KChIP4 [40]. In all our experiments, we expressed Kv4.3/KChIP2 at equal levels.

In our first scenario, the assembly of S390N and ΔF227 mutant homocomplexes, even in the presence of KChIP2, causes partial haploinsufficiency due to almost complete endoplasmic reticulum retention and reduced channel complex stability. Dominant negative effects on the plasma membrane trafficking have also been seen in other potassium channelopathies, such as long QT syndrome type 1 and 2, nonsyndromic sensorineural deafness type 2 and SCA13, which are disorders caused by truncating and missense mutations in other voltage-gated potassium channels Kv7.1, Kv11.1, Kv7.4 and Kv3.3, respectively [41–44]. The dominant effect on WT protein trafficking exerted by these mutant Kv channels might be due to the existence of a common pathomechanism underlying different Kv-driven diseases induced by temperature-sensitive folding deficits. This mechanism was seen in our work and has been seen by others, including in persistent hyperinsulinemic hypoglycemia of infancy caused by mutant pancreatic ATP-sensitive potassium SUR1/Kir6.2 channels [45] and in long QT type 2 syndrome caused by Kv11.1 channel mutations [46].

In our second scenario, loss of function of all WT/mutant heterocomplexes might occur with low KChIP2 levels, such as decline of expression during aging or due to SCA19/22 mutations. Although some studies have reported on KChIP expression and function in the adult rodent cerebellum [47–50], no aging-related expression studies in human cerebellum have been reported thus far. Since no clear differences were observed in the expression levels of KChIPs in the human temporal cortex of middle-aged or older individuals (data extracted from GEO database IDs: 79781991, 79784404, and 79795297), it remains to be established how far such haploinsufficiency may also contribute to disease.

For the third scenario, we have shown that, in addition to the haploinsufficiency, all mutant Kv4.3 subunits also exhibit dominant effects on WT Kv4.3 channel function, even in the presence of KChIP2, which may contribute to disease onset and progression. This is especially true for T352P, which strongly suppressed current amplitude when this mutant subunit co-assembled with WT Kv4.3. The threonine to proline substitution at position 352 in the pore region is seemingly extremely damaging, and we suspect that this mutation leads to a full collapse of the channel pore and thereby complete inactive channels complexes despite its cellular localization. Consequently, WT/T352P heterocomplex channels exhibited significant changes in their time to reach peak amplitude and their *I*_150ms_/*I*_peak_ ratio compared to WT alone, indicating that a limited number of mutant subunits were able to be incorporated into active, cell surface channels. These results indicate that incorporation of one T352P subunit in an otherwise WT channel was not sufficient to abolish channel activity in oocytes, although the suppression of the current amplitude is dose dependent. Furthermore, dominant alterations in gating of the heterocomplex Kv4.3 channel were observed, since the activation and inactivation shifts of all WT/mutant heterocomplexes, except for WT/T352P heterocomplexes showed values between the WT and the mutant homocomplexes. Dominant suppression of channel activity and altered gating were also described for many of the mutant Kv3.3 heterocomplex channels underlying SCA13 [27–29].

All SCA19/22 mutations may modify the outward A-type potassium currents in neurons, leading to changes in the interspike interval of action potentials in Purkinje cells and other cerebellar neurons, such as basket cells or stellate cells that might cause neuronal degeneration similar to the mechanism observed in SCA13 [30]. Since there are no in vivo disease models for SCA19/22, we assume that loss of intrinsic pacemaking in Purkinje cells could underlie motor dysfunction and ataxia, as has been seen in episodic ataxia type 2 mice [28]. The exact role of Kv4.3 in the interspike interval in Purkinje cells remains unknown [51], but some of the gating deficits, including the shift of *V*_½,inact_ in the hyperpolarized direction of the WT/S390N heterocomplexes and the reduced activity of the WT/T352P heterocomplexes (as was seen for a Kv4.2 dominant negative mutant [52, 53]), may both lead to shorter interspike intervals and bursts of action potentials.

Our data clearly show the complexity of establishing a common pathological mechanism underlying SCA19/22, a complexity consistent with the clinical heterogeneity of this disease. Based on our results, we can only establish a relationship between the loss of function of the T352P mutant channel and a more severe phenotype and an earlier age of disease onset in patients, and between channel gating deficits caused by M373I, S390N and ΔF227 and milder ataxic symptoms. However, future work should prove whether SCA19/22-induced alterations in synaptic activity are associated with reduced neuronal survival and will reveal the role that these alterations play in neuroprotection [54].

## Electronic supplementary material

Below is the link to the electronic supplementary material.

Supplementary material 1 (DOC 11756 kb)
