# Supplementary material for: Spinocerebellar ataxia type 19/22 mutations alter heterocomplex Kv4.3 channel function and gating in a dominant manner
Source: Cell Mol Life Sci. 2015 Apr 9;72(17):3387–99. doi: 10.1007/s00018-015-1894-2 (PMC4531139; doi:10.1007/s00018-015-1894-2)
Supplement: Supplementary file 1 — Supplementary material 1 (DOC 11756 kb) [file 18_2015_1894_MOESM1_ESM.doc]

**Supplementary material**

**Spinocerebellar ataxia type 19/22 mutations alter heterocomplex Kv4.3 channel function and gating in a dominant manner**

Anna Duarri^1^, Meng-Chin A Lin^4^, Michiel R Fokkens^1^, Michel Meijer^2^, Cleo JLM Smeets, Esther AR Nibbeling^1^, Erik Boddeke^2^, Richard J Sinke^1^, Harm H Kampinga^3^, Diane M Papazian^4^, and Dineke S Verbeek^1,*^

^1^Department of Genetics,

^2^Department of Medical Physiology,

^3^Department of Cell Biology,

University of Groningen, University Medical Center Groningen, 9700 RB Groningen, The Netherlands.

^4^Department of Physiology, University of California at Los Angeles, Los Angeles, CA 90095-1751, USA.


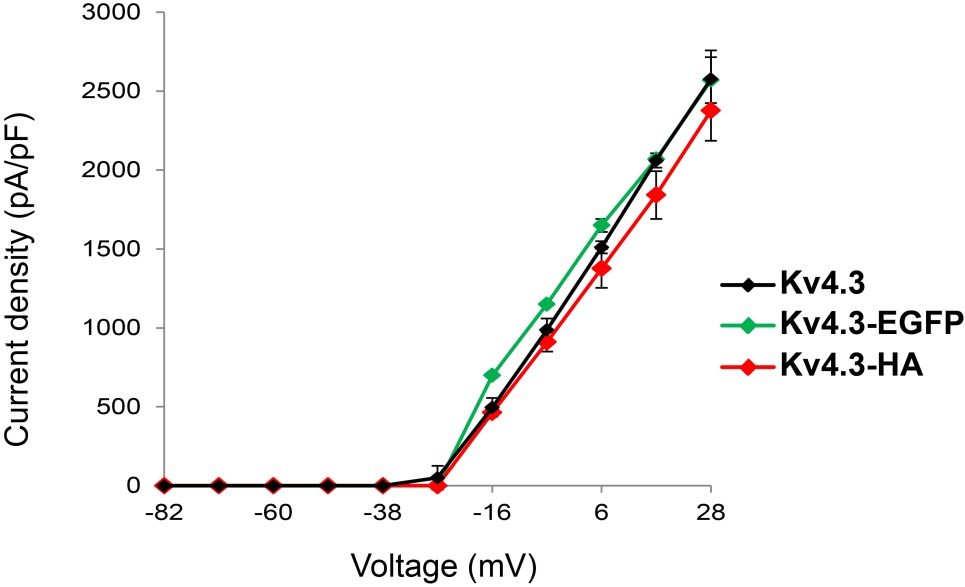


**Figure S1. Comparison of channel activity between Kv4.3 WT, HA-tagged at the first extracellular loop and EGFP-fussed in the C-terminus intracellular tail.**

Potassium currents were evoked by step depolarization of 200-millisecond duration from a holding potential of -82 mV to +28 mV in HEK293 cells transfected with Kv4.3 WT, HA-tagged Kv4.3 WT, and EGFP-fused Kv4.3 WT in the C-terminus, and the current–voltage relationship plotted. All values shown in the graph are the average of at least 10 independent measurements, and the error bars represent the standard error of the mean.

**
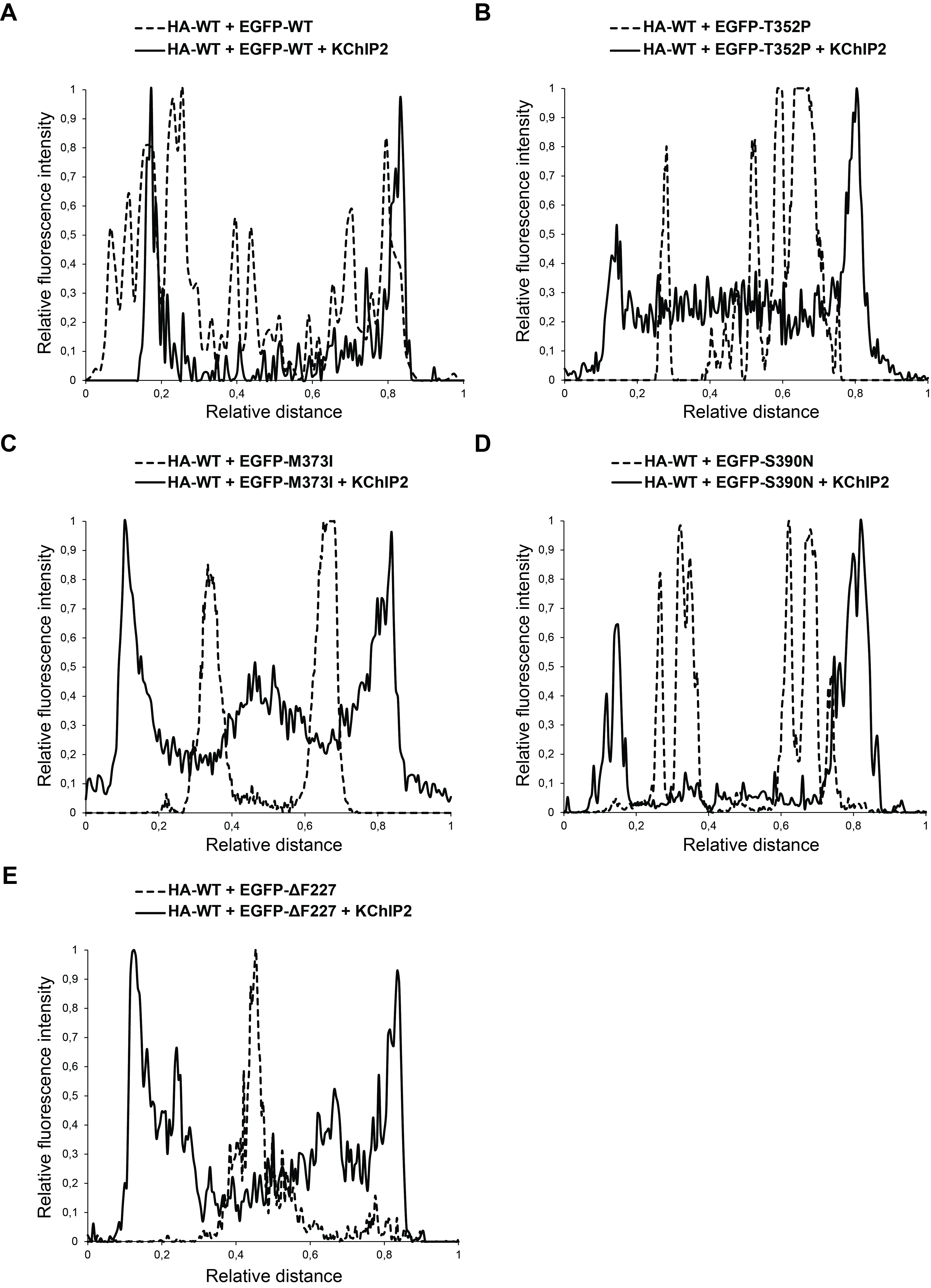
**

**Figure S2. Cellular fluorescence intensity profiles for Kv4.3 WT/mutant heterocomplexes in absence or presence of KChIP2.**

The average fluorescence profile is measured using ImageJ software and the cell edge is determined manually and normalized. Representative plots of intensity profiles plotted as a function of relative distance versus relative intensity (measured in gray-scale values) of images in Figure 1A (dotted line; Kv4.3 WT + mutant) and Figure 3A (black line; Kv4.3 WT + mutant + KChIP2). The graphs clearly show that the fluorescent signal of each Kv4.3 WT/mutant heterocomplexes in presence of KChIP2 is displaced towards the plasma membrane.

**
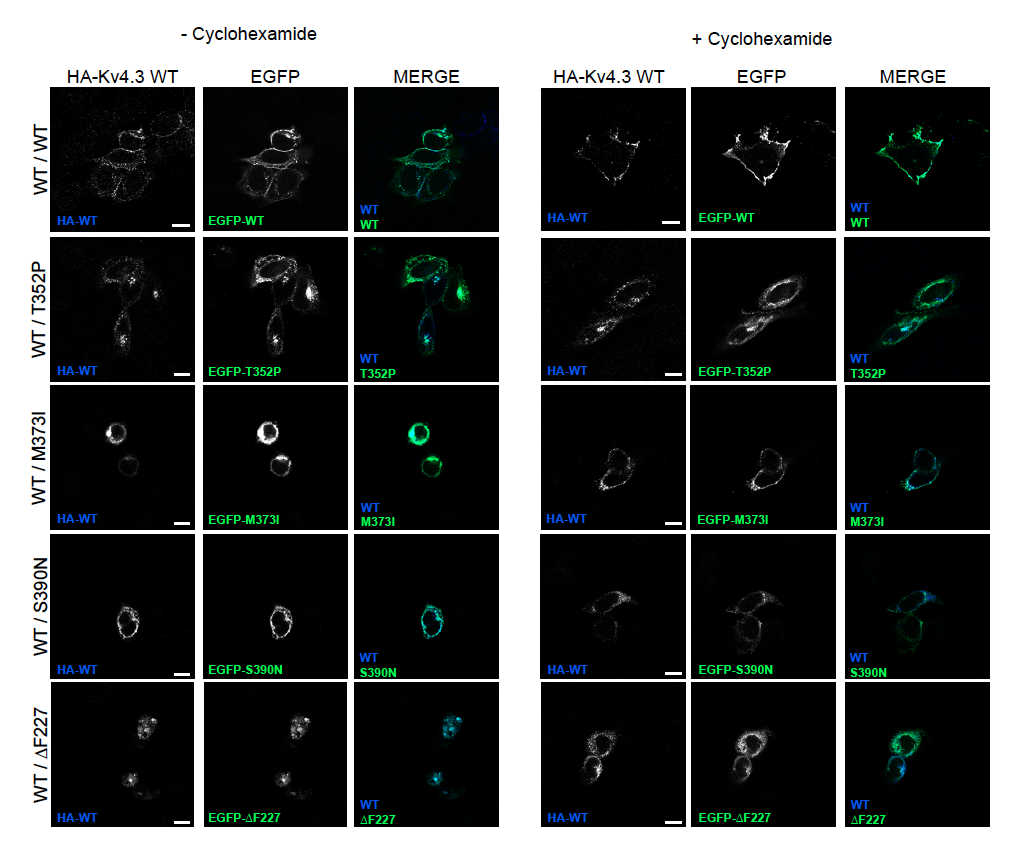
**

**Figure S3. Cellular localization of the various heterocomplex Kv4.3 channel complexes in the presence and absence of cyclohexamide**

Confocal images showing an anti-HA immuno-staining of permeabilized and fixed HeLa cells co-expressing HA-Kv4.3 WT (blue) and EGFP-fused WT, -T352P, -M373I, -S390N or -ΔF227 Kv4.3 (green) in the absence or with cyclohexamide (treatment 6 hours; 25μg/ml). Scale bar = 20 µm. No changes in the localization of any of the heterocomplex channels was observed upon cyclohexamide treatment.

**
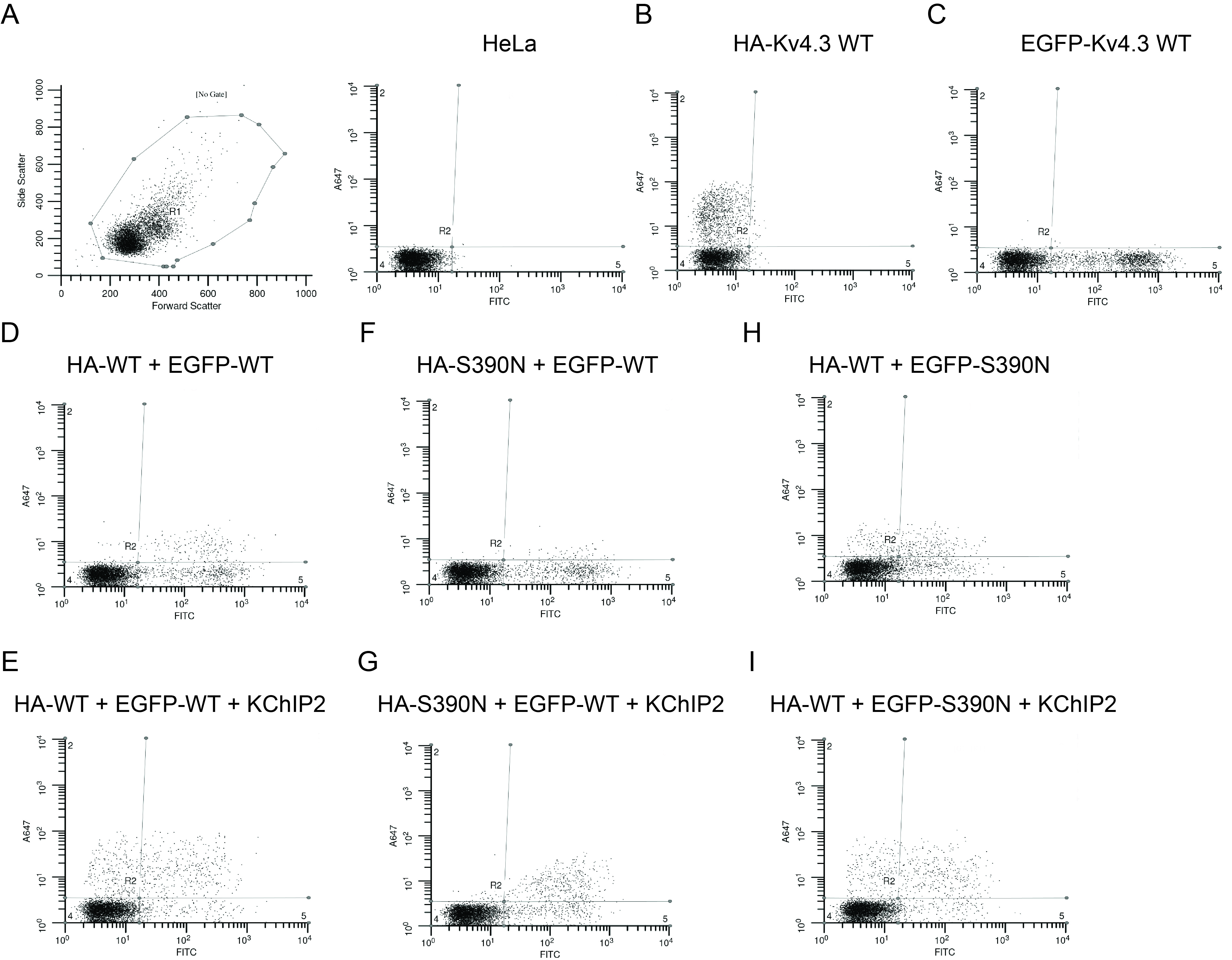
**

**Figure S4. Representative FACS plots to quantify the number of transfected cells expressing extracellular HA-tagged Kv4.3 WT at the plasma membrane.**

Non-permeabilized transfected Hela cells were incubated with anti-HA (Alexa647) to detect Kv4.3-HA at the cell surface of transfected cells (FITC). (A) Non-transfected Hela cells were used as a negative control. (B) Cells expressing HA-Kv4.3 WT was used as a positive HA A647 control and (B) EGFP-Kv4.3 WT as a FITC control. Example of cells expressing HA-WT + EGFP-WT without (D) or with KChIP2 (E) compared with cells expressing either HA-S390N mutant + EGFP-WT (F) or HA-WT + EGFP-S390N mutant (H) both without (G) and with KChIP2 (I). Quantification of surface HA-Kv4.3 was calculated as a percentage of positive A657/FITC cells respect the total FITC (transfected) cells.


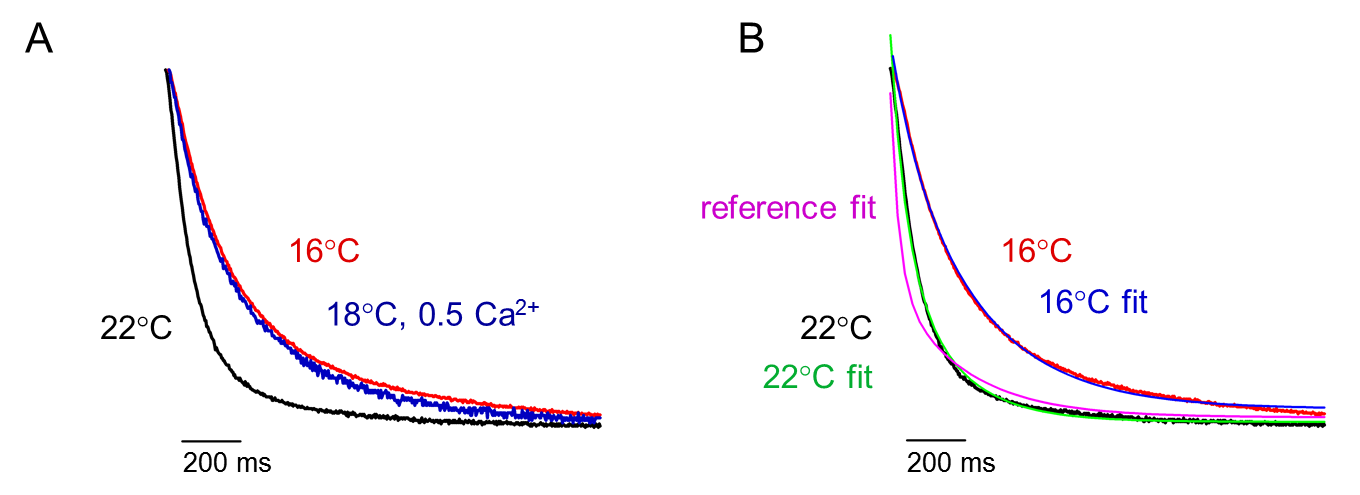


**Figure S5.** **Effect of temperature on Kv4.3 inactivation kinetics.**

RNAs encoding WT Kv4.3 and KChIP2b were injected into Xenopus oocytes at a 1:1 ratio. A) Traces were recorded at 16°C (red) or 22°C (black) by pulsing from -100 to +50 mV for 1 s. Temperature was controlled using a Peltier device and measured with a thermistor. Representative traces have been scaled to the same amplitude and overlaid. The bath contained 2 mM KCl, 98 mM NaCl, 1.8 mM CaCl_2_, 1 mM MgCl_2_, 5 mM HEPES, pH7.5. For comparison, a trace recorded at ~18°C in the 0.5 mM CaCl2 bath solution used in the experiments described in the main text is also shown (dark blue). Note the similarity to the 16°C trace. B) The kinetics of inactivation were fitted with the sum of two exponential components to estimate τ_fast_ and τ_slow_, and their respective amplitudes, A_fast_ and A_slow_. Values for τ_fast_, τ_slow_, and A_fast_ are provided in Table S1. Fits are shown superimposed on the current traces from part A: 16°C trace (red), 16°C fit (blue), 22°C trace (black), 22°C fit (green). Also shown is a two component fit (magenta) from ref. 1 to the inactivation of channels composed of Kv4.3 and KChIP2b at 22°C using the same bath solution.

**Table S1: At 16°C or ~18°C, a slow component of inactivation dominates the kinetics of current decay^1^**

|  | 22°C | 16°C | ~18°C  (1.8 mM Ca^2+^ bath) | Fit from ref. 1  (22°C ± 2°C) | ~18°C  (0.5 mM Ca^2+^ bath) |
| --- | --- | --- | --- | --- | --- |
| τ_fast_ (ms) | 51±2 (12) | 72±4 (10) | 44±1 (9) | 29 | 49±3 (6) |
| τ_slow_ (ms) | 182±14 (12) | 251±23 (10) | 251±8 (9) | 239 | 255±6 (6) |
| A_fast_ | 0.48±0.05 (12) | 0.17±0.04 (10) | 0.15±0.03 (9) | 0.64 | 0.15±0.03 (6) |

^1^ At 16°C or ~18°C, a slow component of inactivation dominates the kinetics of current decay. Currents were recorded at the indicated temperatures by pulsing from -100 to +50 mV for 1 s. The bath contained 2 mM KCl, 98 mM NaCl, 1.8 mM CaCl_2_, 1 mM MgCl_2_, 5 mM HEPES, pH7.5. Inactivation kinetics were fitted by the sum of two exponential components at 16°C, ~18°C, or 22°C as described in the Figure S4 legend. Values for τ_fast_, τ_slow_, and A_fast_ are provided as mean ± SEM. The value of n is provided in parentheses. For comparison, values inferred from ref. 1 and determined from our data obtained at ~18°C in the bath solution containing 0.5 mM CaCl_2_ (see Methods section) are also provided. At 22°C, fast and slow components of inactivation make similar contributions to the overall kinetics of current decay. In contrast, at 16°C or ~18°C, the slow component of inactivation dominates.

**References**

1. Amadi CC, Brust RD, Skerritt MR, Campbell DL (2007) Regulation of Kv4.3 closed state inactivation and recovery by extracellular potassium and intracellular KChIP2b. Channels 1, 305-314.
